# The Conservative Treatment of the Pulp

**Published:** 1884-02

**Authors:** James G. Palmer


THE
AMERICAN JOURNAL
OF
DENTAL SCIENCE.

VOL. XVII. THIRD SERIES—FEBRUARY, 1884. NO. 10

ARTICLE I.

### THE CONSERVATIVE TREATMENT OF THE PULP.

BY JAMES G. PALMER, D. D. S.

What is the Conservative Treatment? what does it mean? and what is it opposed to? are questions likely to be asked concerning such a subject. Webster defines the word conservative as meaning, among other things, "to save, to protect—*that which keeps from injury or* RADICAL *change.*" Consequently it signifies more than the word "preservative," and hence the "conservative treatment" in dental operations, must signify more, embrace different treatment, and secure different and more complete results, than that which is designated simply "preservative."

In speaking of the treatment of the pulp, we necessarily presuppose its exposure, or injury in some way, thus giving rise to a need, either of treating it or of destroying it, and the "conservative treatment" means its preservation if possible, as opposed to its devitalization, which may properly be considered the "radical treatment." I may then speak of the feasibility of the "conservative treatment," of the methods of treatment, and of its superiority.

First, let us glance at the tooth structure, and the relation the pulp bears to the dentine. What is the pulp? It has been described as the " soft vascular part of the tooth within the central chamber," which, though true enough, does not give a definite idea of its relation to the tooth, nor how it became placed within its sheath of dentine and enamel. From without, inward, we have enamel, living membrane, dentine, pulp. The enamel is dense and hard, the dentine somewhat softer, the pulp very soft, being composed of capillary blood-vessels and nerve filaments, held in the meshes of connective tissue, which nerve filaments enter and ramify the tooth structure.

It is not my intention to describe in detail the formation, " in embryo," of the teeth, for we are, or should be, more or less familar with it. But I desire to refer to some of the changes that occur. While the enamel is being formed from the enveloping sac, at the proper period, begins the formation of the dentine from the papilla, or tooth germ itself. As this formation progresses, the papilla is encroached upon, and grows smaller and smaller, as more dentine is formed from it, until the point of completion is reached, when by some law of nature, the process ceases, and the dentine being completely formed, we have a cavity within, filled with a highly sensitive pulp, the " soft vascular part of a tooth " mentioned before, which is the remainder of the papilla, and consists of blood-vessels and nerve filaments, similar to the ordinary papilla of touch. During this time, the formation of enamel has progressed, and the tooth being completed, in due time we have its eruption.

The enamel, dense and hard, is built up of bundles of rods, called " enamel rods," which hold a very small quantity of living matter in the interstices between them. Some eminent microscopists and observers ever finding this living matter in dire contact and communication with the dentine at the living membrane of the tooth, and the name, " enamel fibres," has been given to this substance. In the dentine we find canaliculi, or dentinal tubuli, which it is certain contain " dental fibres," which are offshoots of the pulp, and establish communication from the pulp to every part of the dentine, and in all probability, throughout the enamel also. Necessarily then, any exposure of the nerve, even congestion without its exposure, may result in its death, and the removal of all living matter from the dentine and enamel; and we know that such teeth are more brittle than living teeth.

I have said that the dentine was formed from the papilla. It has been found that as old age comes on, the walls of the pulp-chamber seem to encroach upon the pulp, which grows smaller. Evidently new dentine, or something akin to it, is formed from the pulp, as at an earlier period. Sometimes excresences will be found within the pulp-chamber encroaching upon the pulp. This new formation is called "secondary dentine," and it is certain, that under favorable circumstances, it will be formed from the pulp. If then, the papilla could build up the dentine, and if, in after years, it has still the dentine, it certainly has the power of building up new material after it *does* possess this formative power, and in so much, the "conservative treatment" is certainly feasible: as to the method: The exposed pulp must be carefully protected—put into a healthy condition if need be, so that it may build up this layer of secondary dentine.

In persons in a debilitated condition, suffer ng from systemic trouble, or constitutional difficulty, it may be useless to attempt it, as the pulp, being a part of the system, it is necessarily affected, more or less, by any disturbance of the functions of life. Even in such cases, thorough medical treatment may enable the system to recuperate enough for the benefit of the pulp.

In the case of a person in an ordinary state of health, an attempt may be made to preserve the pulp alive, with almost a positive assurance of success. The cavity in the first place must be thoroughly and carefully cleansed—all debris and broken-down tooth structure being removed. It is then necessary to ascertain the condition of the pulp. If there is merely excitation, with very little or no inflammation, we may proceed at once to stimulate it to action. But if there exists some degree of congestion and inflammation, it will be necessary to use some local application, to reduce and allay it. In *slight* congestion, an application of lead-water, or of aconite and iodine will be efficacious. In *severe* inflammation, it will be well, in addition, to use counter-irritants, hot foot-baths, and possibly internal remedies. By such means, we endeavor to allay the inflammation and bring tooth into the condition first mentioned. A mendicant should now be applied, which, while stimulating the pulp to action, should also act as a sedative, and tend to prevent inflammation. Oil of cloves, creosote, carbolic acid, chloroform aconite and laudanum, are among the agents often used.

The method that has best pleased me is about as follows: After the thorough cleansing spoken of, dress the exposed pulp carefully with glycerine and arnica, or glycerine and calendula. Then, mixing some oxide of zinc with the drug to be used (preferably dilute carbolic acid), to a thick paste, apply it directly to the pulp and over a large portion of the cavity. Carefully absorb any excess of liquid, and see that the edges of the cavity are all free and clean. Then carefully fill up the cavity with any plastic material, but preferably phosphate of zinc, avoiding as far as possible any pressure upon the pulp. Too much stress cannot be put upon two things: the careful preparation of the cavity and extreme care in avoiding pressure upon the pulp. Allow this dressing to remain in the cavity four or five weeks. Then carefully remove the filling, and wash the cavity out gently with tepid water. If progressing favorably, there will be found over the pulp a soft, velvet-like layer, showing, sometimes to the naked eye, fine granulations. This will be soft and thin or thick and hard, as more or less time has elapsed. The dressing should be again applied and sealed as before, and a few weeks more allowed to elapse. On the occasion of the second opening, if the process has continued, it will be safe, using some non-conductor over the new deposit, to proceed with permanent filling.

There are some variations in the result. Of course, after all care, the pulp may die; but the percentage of loss is not large, and if the pulp does die, it will be a painless death in most cases. Again, at the time of the first opening, it may be found that apparently there has been no change. The application should again be made, with such changes in the drug as experience may have taught; and if, at the second opening, there still seems to be no change, the dressing may be again applied, and with some thin metal cap placed over it, to prevent pressure, the cavity may be permanently filled, By such a method, a pulp may be kept a long time without even forming secondary dentine, and some assert their ability to preserve the nerve in one or two roots, when the third root may have been devitalized.

Lastly, as to the superiority of the "conservative treatment." It preserves the tooth, and restores it to its full strength, as far as the supply of nutriment is concerned. It keeps the healthful, glistening, life-like appearance of the whole tooth. It gives to the tooth, by reason of the filaments of living matter ramifying throughout its structure, greater ability to resist decay and to resist fracture. It prevents all danger of trouble from abscesses. On the other hand, the "radical method," the devitilization of the pulp, leaves the tooth shorn of its strength, its nutriment being gone; it loses its glistening, life-like appearance, and becomes dull and opaque—in fact, a dead tooth. Greater than all, by reason of the removal of the living matter from the canaliculi and tubuli, the tooth is more brittle, and will not stand such pressure as a living tooth.

Certainly, then, that treatment which preserves the life, color and strength of the tooth is to be preferred, and it seems to me that it is our duty to do the best we can to preserve the life of the injured, sick or inflamed pulp, when presented to us for our consideration, for while there is life there is hope.

Dr. Watkins—I think the paper is, on the whole, an excellent one. I think the preservation of the pulp is one of the grandest things in dental surgery. Of course, in some cases it is impossible, I suppose, to save the pulp, at least in the hands of most of us, but in most cases I think the pulp can be saved. He speaks of some one saving the pulp in two roots, when one was already dead. I think even that can be done. I have seen cases in my own practice where, after having one root filled, in the molars, the nerves appear to be alive in the others after two or three years' standing; and I think it good practice not to kill them; I think we stand a chance of getting better results by not killing than by killing them. A capping can be put on an ordinary exposed nerve, and even where it is a little more than ordinary it can be done. I have a case that I capped a year and four or five months since. It was a lower wisdom tooth that had been condemned by several dentists in New York, four years before the lady came to me; it was entirely broken down, and the upper wisdom tooth had worked its way down so that it forced its way and fitted itself into the cavity of the lower one. In the first place the centre was enlarged a little, so that it bulged up or rounded up out of the pulp-chamber. I made a saucer-shaped cap of hard rubber, filled the cap with vaseline, placed it right over the nerve and then filled the tooth with amalgam. The filling had to be so thin, after putting on the cap, that at one spot the rubber was exposed. About a month ago the patient was in my office, and as the amalgam was wearing away a little, I took a corundum wheel and cut away some of the grinding surface of the upper wisdom tooth, so as to have a chance to put more amalgam on the lower one that I had so treated; and in making the retaining points for the filling I cut into the dentine of this tooth, and I found that it was then as sensitive and as alive as any tooth you ever saw. That was a year and about five months after I had capped the pulp and filled the crown.

Dr. Mills—In connection with this question of the conversation of the pulp, I am reminded of a little circumstance that took place in the Brooklyn Dental Society some sixteen years ago. A very worthy man in our profession then undertook to pass a resolution condemning the barbarous practice of attempting to conserve the pulp by capping, or otherwise. That man has lived to see the folly of such a resolution. The practicability of the conservation of the pulp is a foregone conclusion. No sane man now doubts that at all. But, it is interesting to look back and see the severe ordeal that has had to be gone through in bringing this matter to the standard we find to-day. Our ideas regarding the vitality of the pulp, and its possibilities under treatment, have advanced a great deal. We find that we can not only conserve an exposed pulp when we have the whole of it to work upon, but that we can conserve it partially, or the part that may be remaining. I think Dr. Atkinson has been foremost, perhaps, in promulgating this idea of the conservation of the pulp. I had a case in my practice which shows the possibilities of the pulp. I first noticed this case three years ago, next October. It was a lateral incisor which had been in my hands before, and had been filled ; it had been broken off, and when it came to me I found the head of the pulp had sloughed away, and that the remainder of the pulp was living. The patient was twenty-four years of age. I covered the pulp temporarily and made another appointment. I cut away a portion of the pulp to make space enough to put the proper amount of pivot, and applied an artificial crown to that root, and that pulp is living to-day, and is just as healthy a pulp as any pulp could be under the circumstances. It is an isolated case in that particular. It simply shows the possibilities of the pulp under proper treatment, but it is enough to show that the tendency of our practice is upward in this direction. And I predict that the time is coming when a great many good men who, to-day, persist in using arsenic, will be ready to see themselves the sin and folly of it. We are coming to understand with greater certainty, the structure of the teeth and their relations to the general organization, and as fast as men become intelligent in that matter, they will " right about face." A man who will persist in the destruction of the pulp, in view of the demonstrated possibilities of its preservative treatment, is either weak or wicked. When I say weak, I mean not intelligent, and I like to see anything that has a tendency to open their eyes. It is not an exaggerated statement to say that hundreds and thousands of people are carrying abscesses in their mouths to-day, that may be traced back to the destruction of a pulp.

Dr. Brown—I think we do not generally understand the pulp as an organ at all. It does a great many queer things. I had a case in our office some time ago, that of a young boy, one of this fidgety kind, who had a six-year molar that was troubling him very much. I could not put an instrument in the tooth to clean it out at all. It was a tooth that would eventually have to come out in order to make room, so I decided to kill the nerve as the only thing to do. The boy was suffering from toothache. and I could not do anything with it permanently. I applied a preparation to kill it, and supposed it did kill it, for I succeeded in putting a broach down each of the roots once, and getting out what I supposed was the most of the pulp. After that I could not get the instrument in at all, as his mouth flew shut like a vice. I put a piece of cotton and sandarac in the tooth, with a little carbolic acid underneath. The boy went off somewhere and was away several months. He came to me on his return, and on taking out this cotton and sandarac, I found that a new pulp had come, or something else that had all the appearance of a pulp, and not a fungus pulp either, but a normal pulp. We treated it some little time and at last I capped it, and it has been going so until the present time and is perfectly well. I have examined it twice since, and found it in good condition and bearing all the characteristics of perfectly normal tissue.

Dr. Osum—I don't like to see this paper passed without my saying a word of congratulation to Dr. Palmer, on his bringing that paper before the Society. I believe a great deal of what he has said, and also a considerable part of what Dr. Mills has said, but there seems to be one fact that is overlooked in all these papers and discussions, in regard to conservative pulp-treatment, and that is the condition of your patient and the organic structure of the tissues. There are some very queer and strange things sometimes presented to us. Sometimes you will find a tooth in which the pulp cavity is large and the pulp has been exposed for two, three, or four years, and but little inflammation going on, and it has never developed into anything beyond that. Now, it strikes a man who observes that, that if that tooth had healthy surroundings that pulp could have been preserved. I do not doubt it at all. But I have known cases where the pulp had been covered carefully, and all the skill of eminent practitioners brought to bear to preserve it, and they say, in a year's time that it is a perfect success. But a year's time is no test of the life of a pulp. Wait until five, six, eight, or nine years have gone by, and read its history, and oftentimes it will be a history of the dead. And I don't believe in covering every candidate that presents itself. I believe a great many teeth are better devitalized. The reason that some fail to save a tooth after devitilization is that the operation is not properly done. If the pulp is thoroughly removed and its cavity properly filled to the end of the root, there is not much danger of abscess. Oftentimes we find a patient that has been troubled with an ulcerated tooth, so-called ; the pulp is carefully covered, and has had pus within its borders for three or five years, according to the condition of the patient ; it goes on, and after a while we find a dead pulp has been there, which we considered a live one, and you have an abscess. It is harder to save an abscessed tooth than it is to save one not abscessed. Then, there is another fact, that as people grow old the pulp diminishes in size, sensitiveness and importance. I have taken teeth from the mouths of old persons, in which the pulp-chamber was so small and the pulp so shrunken that you would hardly believe it possible for it to be a live pulp. This shows, that after a tooth is once fully formed, the pulp is to a large extent, superfluous, having performed the functions for which it was created, and after the depositiom of the lime salts has taken place, you can sometimes dispense with it and have admirable results.

Dr. Palmer—I am afraid you misunderstood me. I said that, after the first opening, under certain circumstances, it might be found that there was no change in the pulp, and then it might be covered a second time, a little cap placed over it, and the tooth filled. I did not advise that treatment in all cases, but said it might be done. The idea of my paper was, that when a nerve is found in a proper condition for treatment, it should be preserved. Some care must be taken to ascertain the condition of the pulp by opening it; it must be carefully covered for a certain time, and after the lapse of that time, when you open it, you will find that a layer of new dentine has been deposited over the pulp; and after it has progressed further, and you again uncover it, you will find that the layer of dentine has increased and hardened, and is sensitive. I have had practical proof of this. This treatment does not always succeed. The pulp should be covered temporarily with a non-conductor, and opened from time to time for examination, until you are satisfied that a layer of dentine has formed. Then you may safely proceed to fill the tooth permanently without any danger of abscess afterward.

Dr. Baldwin—One idea about the pulp-treatment I did not fully understand. It was that in some cases, as persons came to be older, there was a secondary dentine formed from the pulp itself—that the pulp had a tendency to build up secondary dentine. As I understand that matter, and as I know it is stated in Tomes' celebrated work, only germs, similar to itself in nature, can be produced by the pulp. The pulp will produce germs of its own nature, then it will produce corpuscles of dentine, and in that case it will be filling up the pulp cavity and encroaching on the pulp itself. A seed of one kind will not produce another kind, and if you want to have dentine you must have it from germs of dentine.

Dr. Kingsley—I think we require a great deal of patience in treating these teeth with their pulps exposed, but I think that with a proper exercise of patience we shall finally be able to fill those that have exposed nerves without any protection whatever. I have in mind the case of a girl who came to me; she was a niece of ex-President Grant. I had great interest in her case. Her teeth decayed very early. I found a lateral incisor with an exposed nerve. I washed it out with carbolic acid, wiped it perfectly clean, and put in an oxy-chloride filling or phosphate, I don't know which. I left it so for about six months, at the end of which time she came, and I took out the filling and found the nerve still exposed, but in better condition. I filled it again, and told her to come and see me occasionally for about a year. She did so. I took it out and refilled it again, each time protecting the nerve. About two months ago she came to me, and I thought I could fill the tooth permanently. I took out the filling; there was no exposed pulp there, and I filled it with gold without any trouble at all. I have seen the girl within five days, and she has not had a particle of trouble from that filling.—*Transactions of New Jersey State Dental Association.*

